# An acute functional screen identifies an effective antibody targeting amyloid-β oligomers based on calcium imaging

**DOI:** 10.1038/s41598-018-22979-2

**Published:** 2018-03-15

**Authors:** Xueying Wang, Ksenia V. Kastanenka, Michal Arbel-Ornath, Caitlin Commins, Akira Kuzuya, Amanda J. Lariviere, Grant A. Krafft, Franz Hefti, Jasna Jerecic, Brian J. Bacskai

**Affiliations:** 1 Massachusetts General Hospital, Department of Neurology, 114 16th Street, Charlestown, MA 02129 USA; 2 Acumen Pharmaceuticals, Inc., 4435 North First Street, #360, Livermore, CA 94551 USA; 3 Present Address: Harvard University, Center for Brain Science, 52 Oxford Street, Cambridge, MA 02138 USA

## Abstract

Soluble amyloid β oligomers (AβOs) are widely recognized neurotoxins that trigger aberrant signaling in specific subsets of neurons, leading to accumulated neuronal damage and memory disorders in Alzheimer’s disease (AD). One of the profound downstream consequences of AβO-triggered events is dysregulation of cytosolic calcium concentration ([Ca^2+^]_i_), which has been implicated in synaptic failure, cytoskeletal abnormalities, and eventually neuronal death. We have developed an *in vitro*/*in vivo* drug screening assay to evaluate putative AβO-blocking candidates by measuring AβO-induced real-time changes in [Ca^2+^]_i_. Our screening assay demonstrated that the anti-AβO monoclonal antibody ACU3B3 exhibits potent blocking capability against a broad size range of AβOs. We showed that picomolar concentrations of AβOs were capable of increasing [Ca^2+^]_i_ in primary neuronal cultures, an effect prevented by ACU3B3. Topical application of 5 nM AβOs onto exposed cortical surfaces also elicited significant calcium elevations *in vivo*, which was completely abolished by pre-treatment of the brain with 1 ng/mL (6.67 pM) ACU3B3. Our results provide strong support for the utility of this functional screening assay in identifying and confirming the efficacy of AβO-blocking drug candidates such as the human homolog of ACU3B3, which may emerge as the first experimental AD therapeutic to validate the amyloid oligomer hypothesis.

## Introduction

Soluble amyloid β oligomers (AβOs)/Aβ-derived diffusible ligands (ADDLs) were first shown to be potent neurotoxins nearly twenty years ago^1^, and today, it is widely recognized that AβOs trigger aberrant signaling in specific subsets of neurons, leading to accumulated neuronal damage and memory disorders which occur in Alzheimer’s disease (AD)^1–7^. Endogenous AβOs are elevated in brain tissue from human AD patients and from AD transgenic (Tg) mice^2,3,6,8–10^. It has been clear for some time that synapse loss preceding neuronal death is closely associated with early AD-related memory deficits^11–13^, and emerging evidence now suggests that synapse failure is a distinct consequence of AβOs rather than other Aβ species^12,14–16^. The obvious implication of this evidence is that the most effective intervention strategy is direct blockage of AβOs, their formation, or their aberrant signaling activity.

Over the past decade, tremendous effort has focused on anti-amyloid-β (Aβ) immunotherapy for AD, but to this point, no meaningful clinical benefit has resulted from the Aβ-directed therapeutic antibodies that have completed clinical trials^17–21^. Among the Aβ immunotherapeutics currently in human testing, only aducanumab continues to hold promise for potential clinical success^22^. However, because aducanumab does not discriminate between AβOs and the million-fold more prevalent Aβ fibrils, it is difficult to predict that even high dosing of aducanumab will bring about effective blocking of AβO neurotoxicity.

The common feature of all failed Aβ-directed therapeutic antibodies has been their lack of binding selectivity for AβOs, which are the most potent, neurotoxic form of Aβ. Thus, in spite of the fact that treatment with several of the failed antibodies has reduced amyloid plaque levels based on amyloid imaging with PiB, there has been no statistically significant improvement in cognition, or even significant deceleration of cognitive decline. The critical missing feature is the ability to block synaptic attack by AβOs, or at least to lower brain levels of AβOs in order to reduce the extent of aberrant signaling they trigger. It is imperative that AD-directed drug discovery deploy screening and characterization strategies that identify AβO-targeting therapeutics.

One of the profound downstream consequences of AβO-triggered aberrant signaling is dysregulation of cytosolic calcium concentration ([Ca^2+^]_i_), which is part of the final common pathway for pathogenic changes resulting in synaptic failures, neuronal death, network dysfunction and cognitive impairment^23–25^. Calcium homeostasis is tightly governed by [Ca^2+^]_i_ and dynamic fluctuations or/and chronic elevations in local resting calcium levels can compromise many neuronal functions. We have developed a functional screening and profiling assay that capitalizes on multiphoton imaging to measure [Ca^2+^]_i_ with high sensitivity, enabling real-time characterization of aberrant signaling effects triggered by low, physiologically relevant concentrations of AβOs. This assay enables screening and identification of potential AβO-blocking drug candidates in neuronal cell cultures *in vitro*, and on exposed brain surface cortical neurons *in vivo*. In either deployment, we are able to quantify AβO-induced aberrant signaling by measuring [Ca^2+^]_i_ simultaneously in a large number of neurons. Using this method, together with an immunodepletion assay, we have been able to evaluate the binding sensitivity and selectivity of candidate antibodies that are directed towards particular amyloid species.

Among the potential drug candidates available to us for screening and evaluation was a monoclonal antibody ACU3B3 (hereinafter abbreviated as 3B3), which was highly effective in blocking AβO-induced calcium elevations in neuronal cultures and in wild-type (WT) living animals. 3B3 is the murine precursor of the clinical candidate antibody ACU193^9,10^, which has demonstrated high binding selectivity for AβOs versus either monomeric or fibrillar Aβ (U.S. Pat. Nos.7,811,563 and 7,780,963). We also deployed array tomography, a hybrid technique combining ultrathin sectioning with high resolution 3D optical imaging^26,27^ to characterize the interaction of AβOs (labeled by 3B3) with synaptic proteins in APP/PS1 Tg mouse and human AD brains. Together, these results provide strong evidence that AβO-selective therapeutics offers tremendous potential benefit to AD and early memory-compromised patients.

## Results

### Acute functional assay for drug screening based on calcium imaging *in vitro*

**Figure 1:**
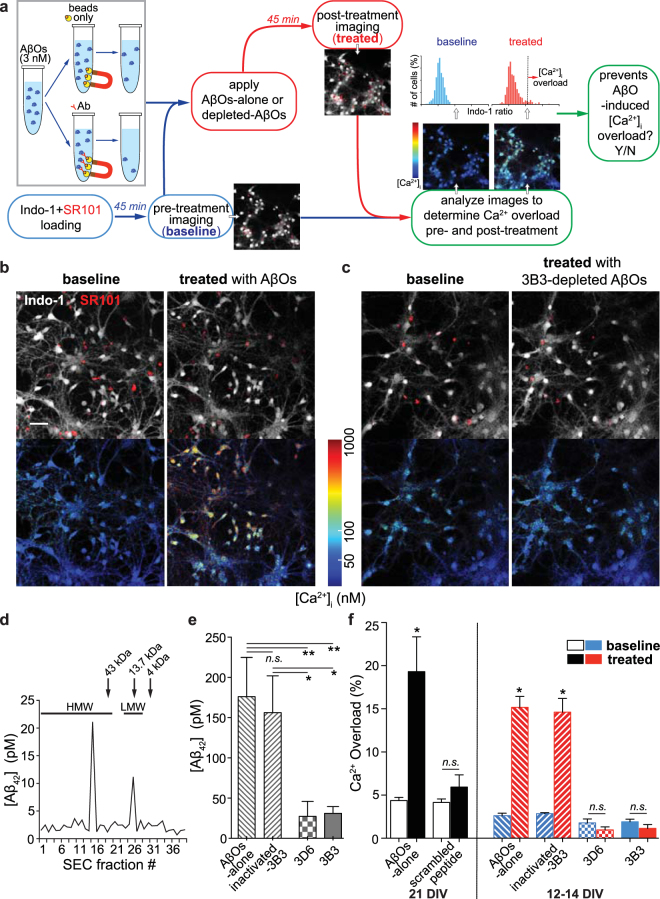
Acute functional assay for initial drug screening *in vitro*. (**a**) Schematic diagram of the functional screening procedures using primary neuronal cultures. (**b**–**c**) Each neuronal culture dish was imaged before (baseline) and after treatment with AβOs-alone or antibody-depleted AβO solutions (treated). Single-channel intensity images (upper panels) reveal indo-1 AM loaded cell bodies (white) in the field of the view with combined labeling of SR101 (red) to differentially identify neurons. Ratios of bound/unbound indo-1 were calculated and converted to absolute calcium concentrations, and images were rendered using a colormap to indicate intracellular calcium levels (lower panels). Incubation of AβOs-alone for 45 min induced significant elevations in intraneuronal calcium levels in a fraction of cells (**b**), whereas baseline calcium was unaffected after being treated with 3B3-depleted AβOs (**c**). (**d**) Biochemical characterization of the synthetic AβO preparation using size-exclusion chromatography (SEC). The molecular weight markers (kDa) ran at the same conditions with the test AβO preparation are indicated above (arrowheads). (**e**) Quantification of Aβ_42_ content detected in the supernatant of AβOs-alone and antibody-immunodepleted AβO solutions using ELISA (n = 6 batches for each condition; two-tailed Mann-Whitney *U* test, *p < 0.05, **p < 0.01). (**f**) Percentage of neuronal cell bodies exhibiting calcium overload before (white bars) and after treatment (black bars) with AβOs-alone or a scrambled control peptide in 21 DIV primary cultures (left panel; n = 6 dishes for each treatment group; two-tailed Mann-Whitney *U* test, *p < 0.05). Intraneuronal calcium overload fractions of pre- (blue bars) and post-treatment (red bars) for four treatment groups using AβOs-alone, heat-inactivated 3B3-, 3D6- and 3B3-immunodepleted AβO solutions in 12–14 DIV cultures, respectively (right panel; n = 6 dishes for each treatment group; two-tailed Mann-Whitney *U* test, *p < 0.05). Data are presented as mean ± s.d. Scale bar: **b** = 50 µm, applies to all images in (**b**) and (**c**).

To assess the ability of anti-Aβ antibodies to target AβOs selectively, we designed an acute assay of AβO-induced neurotoxicity in primary neuronal cultures with [Ca^2+^]_i_ measured as a readout. In our initial survey experiments (Fig. 1a), magnetic beads were coated with an excess of each test antibody (9 µg) to immunodeplete AβOs assembled from 3 nM Aβ_1–42_. Uncoated magnetic beads (i.e. AβOs alone) were used to generate the control preparations for this immunodepletion protocol. After immunodepletion, collected supernatant served as final working AβO solutions for treating the cultured cells. The ratiometric calcium indicator indo-1 was used for intracellular calcium imaging, and the astrocytic marker SR101 permitted differentiation between astrocytic and neuronal calcium signals. After baseline imaging (pre-treatment), the cultures were treated with either control AβO working solutions or solutions immunodepleted of AβOs by various test antibodies. The pre-treatment regions in each culture dish were located and reimaged to measure the post-treatment [Ca^2+^]_i_. Neuronal cell bodies in images obtained before and after treatment were selected and analyzed (detailed analyses are described in the Methods section) to determine the fraction of cells exhibiting calcium overload after treatment. Antibodies that effectively blocked the AβO-induced calcium elevations were designated for further validation.

Among the eight antibodies we tested in primary neuronal cultures, 3B3 along with 3D6 exhibited excellent AβO-blocking capability *in vitro*. Neuronal cultures treated with the AβOs-alone solution exhibited a notable increase in [Ca^2+^]_i_ in a sub-population of neurons (Fig. 1b), whereas cultures treated with vehicle (data not shown) or with 3B3-depleted AβO solutions (Fig. 1c) left the baseline [Ca^2+^]_i_ unchanged.

AβOs assembled from synthetic Aβ_1–42_ have been demonstrated to be stable, reproducible and structurally equivalent to AD brain-derived AβOs, with strikingly similar binding properties to neuronal compartments^3,6,28^. For this assay, we deployed a modified low concentration AβO preparation, similar to the lower concentration preparations published by Rammes *et al*.^29^. In this low concentration method, assembly of 1–5 nM Aβ_1–42_ monomer occurs at 37 °C, giving rise to AβO solutions with relatively high specific activity, compared with the ADDL preparations typically assembled from 100 µM Aβ monomer that are used in many published studies^30,31^. Size-exclusion chromatography (SEC) and Aβ-specific ELISA were used to characterize these AβO preparations. These methods confirmed that the higher molecular weight (HMW) AβOs were considerably more prevalent than low molecular weight (LMW) Aβ species (Fig. 1d). The SEC characterization also indicates that the mean size and dispersity of larger AβO peak is considerably smaller than the HMW SEC fraction from a typical ADDL preparation, as characterized by Hepler *et al*.^32^, which exhibited a mean MW of 223 kDa, in a range from 150 to 1,000 kDa.

Our subsequent experiments involved the following four treatment conditions: AβOs-alone, heat-inactivated 3B3-, 3D6-, and 3B3-immunodepleted AβOs, respectively. Among these different solutions, the final measured concentrations of Aβ_1–42_ in the AβOs-alone or heat-inactivated 3B3-depleted solution were 176 ± 49 pM and 156 ± 46 pM, respectively. In contrast, Aβ_1–42_ concentrations in the 3D6- and 3B3-depleted AβO solutions were considerably lower, 27 ± 19 pM and 31 ± 8 pM, respectively. (Fig. 1e; n = 6 batches for each condition; mean ± s.d.; Mann-Whitney *U* test, *p < 0.05, **p < 0.01). Notably, the 15-fold drop from 3 nM for the initial AβO solution following incubation with uncoated magnetic beads likely results from non-specific binding to the beads or the Eppendorf tubes used for incubation^33^.

Using the pre-determined dissociation constant (K_D_) and the dynamic range of the indicator obtained from *in vitro* calibration experiments (see Methods), indo-1 ratios were converted into absolute calcium concentrations. The data obtained indicate that [Ca^2+^]_i_ is tightly regulated in WT neuronal cultures at an average level of 40 ± 14 nM (mean ± s.d.; n = 4,867 cells; Shapiro-Wilk normality test, *p* = 0.394) at 12–14 DIV and 39 ± 18 nM (mean ± s.d.; n = 2,673 cells; Shapiro-Wilk normality test, *p* = 0.659) at 21 DIV. At rest, ~2% of the cultured neurons exhibit calcium overload at baseline at 12–14 DIV (Fig. 1f, blue bars), whereas in 21 DIV cultures the resting overload fraction increases to ~4% (Fig. 1f, white bars). This increase may be attributable to increased spontaneous agonism of synaptic AβO-binding receptors, which are known to be developmentally upregulated^6,34^. When AβOs-alone were applied to 21 DIV cultures, ~20% of the neurons exhibited significant calcium overload, whereas addition of a scrambled control peptide preparation had no effect (Fig. 1f, left panel; calcium overload at 21 DIV: AβOs-alone - before 4.38 ± 0.36%, after 19.31 ± 4.04%, *p* = 0.029; scrambled control peptide - before 4.16 ± 0.39%, after 5.94 ± 1.41%, *p* = 0.66; mean ± s.d.; n = 6 for each condition; Mann-Whitney *U* test). In 12–14 DIV cultures, incubation of AβOs-alone resulted in a prominent increase in baseline calcium to ~13% calcium overload (Fig. 1f, right panel; calcium overload at 12–14 DIV: AβOs-alone - before 2.61 ± 0.29%, after 15.18 ± 1.27%, *p* = 0.028; mean ± s.d.; n = 6; Mann-Whitney *U* test). The extent of AβO-induced increase in [Ca^2+^]_i_ in each neuron is likely dependent on the prevalence of AβO-binding synaptic receptors at a particular AβO concentration, however the fraction of neurons that exhibit calcium overload is not substantially different for 12–14 DIV cultures and 21 DIV cultures, so we chose to conduct all screening using 12–14 DIV cultures to reduce variability and increase throughput. For purposes of studying the underlying signaling cascade, 21 DIV neurons would likely provide much greater sensitivity.

Application of AβOs depleted with heat-inactivated 3B3 induced significant calcium overload similar to AβOs-alone preparations, and this deleterious increase was completely abolished by immunodepletion with 3D6 or 3B3 (Fig. 1f, right panel; calcium overload at 12–14 DIV: heat-inactivated 3B3 - before 2.85 ± 0.12%, after 14.61 ± 1.59%, *p* = 0.039; 3D6 - before 1.78 ± 0.46%, after 0.95 ± 0.35%, *p* = 0.22; 3B3 - before 1.92 ± 0.30%, after 1.15 ± 0.44%, *p* = 0.18; mean ± s.d.; n = 6 for each condition; Mann-Whitney *U* test).

### *In vivo* validation of 3B3 protection from AβO-dependent calcium dysregulation

**Figure 2:**
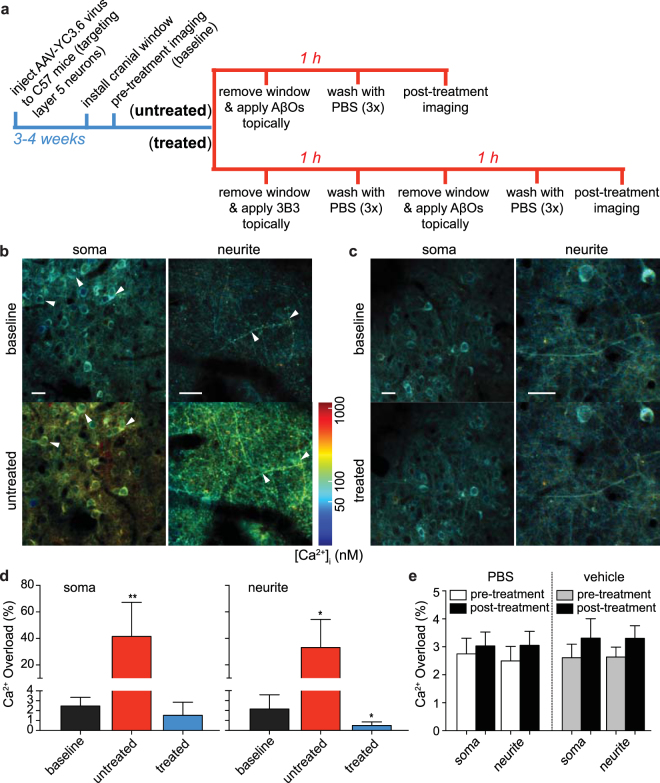
Evaluation of binding selectivity of 3B3 to AβOs *in vivo*. (**a**) Experimental procedure for direct topical application of 5 nM synthetic AβOs (untreated) or acute treatment with 1 ng/mL 3B3 prior to AβO application (treated) onto 3–4-month-old WT mouse brain followed by multiphoton imaging with encoded ratiometric calcium indicator YC3.6. (**b**–**c**) Neurons and neurites filled with YC3.6 in cortical layer 5 were imaged before (i.e. baseline; upper panels) and after (lower panels) topical applications (n = 6 mice for each treatment group). Ratio images were pseudocolored to show correlated calcium concentrations. Exposure of cortical surface to AβOs alone (**b**) elicited significant calcium overload in neuronal somata and neurites (examples are arrowhead-pointed in **b**). In contrast, [Ca^2+^]_i_ of cellular compartments were well maintained in 3B3-pretreated group (**c**). (**d**) Percentage of neuronal cell bodies (left panel) and neurites (right panel) exhibiting calcium overload in untreated and treated groups (number of somata analyzed: untreated before n = 2,316, after n = 2,145; treated before n = 3,726, after n = 3,613; number of neurites analyzed: untreated before n = 1,912, after n = 1,700; treated before n = 1,696, after n = 1,696; two-tailed Mann-Whitney *U* test, *p < 0.05, **p < 0.01). Baseline calcium overload was determined by grouping all the pre-treatment measurements together (black bars; n = 12 mice; individual datasets were compared by Kruskal-Wallis test, *p* = 0.16 for datasets in soma group and *p* = 0.22 for datasets in neurite group). Post-treatment measurements for untreated (red bars) and treated (blue bars) groups are shown separately (two-tailed Mann-Whitney *U* test based on n = 6 mice for each group, *p < 0.05, **p < 0.01). The *y*-axis is continuous (0–80%) but with two different scales. (**e**) Percentage of cellular compartments exhibiting calcium overload after 2 h treatment with PBS (left panel; n = 3) or vehicle (right panel; n = 3). Neither condition induced notable increases in [Ca^2+^]_i_ (Wilcoxon signed-rank test; *p* = 0.108 for all compared pairs). Data are shown as mean ± s.d. Scale bars: all scale bars in **b** and **c** = 20 µm.

Because the initial higher throughput screening was carried out in developing neurons (12–14 DIV), in which AβO-selective receptor density has not reached maximum levels and the full manifold of downstream signaling pathways may not have been fully represented, we sought to evaluate AβO-mediated calcium increases and the blocking ability of 3B3 *in vivo*. To this end, we used multiphoton microscopy (Fig. 2a) to measure changes in neuronal [Ca^2+^]_i_ in adult WT mice that had been virally transduced with the ratiometric calcium sensor YC3.6, targeted to layer 5 neurons in the somatosensory cortex^35^. Imaging was accomplished through a cranial window installed above the injection site 3–4 weeks after viral vector injection. Pre-treatment imaging to establish baseline calcium levels was carried out first, followed by opening of the cranial window and direct application of 5 nM AβOs to the cortical surface. After a one-hour incubation, the brain surface was washed three times with phosphate-buffered saline (PBS), a new coverslip window was installed, and the animal was re-imaged (post-treatment) to determine changes in [Ca^2+^]_i_. A fluorescent angiogram was used as a reference, enabling alignment of pre-treated regions with the post-treatment regions in all the experiments. To assess antibody blocking capability, the cortical surface of the brain was pre-treated with 1 ng/mL 3B3, followed by three PBS washes prior to final exposure of the cortical surface to AβOs.

Pre-treatment measurements showed the average [Ca^2+^]_i_ to be 78 ± 11 nM for neuronal somata (mean ± s.d.; n = 6,042 cell bodies; D’Agostino & Pearson omnibus normality test, *p* = 0.035) and 70 ± 15 nM for neurites (mean ± s.d.; n = 3,608 neurites; D’Agostino & Pearson omnibus normality test, *p* = 0.014) in WT mice. These values were comparable to previously reported *in vivo* measurements of neuritic [Ca^2+^]_i_^35,36^. One-hour acute exposure of naive brains to 5 nM AβOs (Fig. 2b) triggered dramatic calcium elevations in ~40% of neuronal somata as well as ~30% of neurites (Fig. 2d, red bars; soma 41 ± 25%, *p* = 0.0008; neurite 33 ± 21%, *p* = 0.0238; mean ± s.d.; n = 6; Mann-Whitney *U* test). In contrast, no significant elevations in baseline calcium were observed in cell bodies or neurites when 3B3 was applied for one hour prior to AβOs application (Fig. 2c), i.e., the AβO-elicited adverse effect was entirely blocked by 3B3 pre-treatment (Fig. 2d, blue bars; soma 1.52 ± 1.33%, *p* = 0.111; neurite 0.42 ± 0.37%, *p* = 0.0357; mean ± s.d.; n = 6; Mann-Whitney *U* test).

Furthermore, application of either PBS or vehicle to the cortical surface did not trigger significant increases in calcium overload (Fig. 2e), indicating that neither the surgical procedure nor the AβO preparation vehicle produced non-specific calcium elevations.

### 3B3 targets AβOs at synapses in APP/PS1 mouse brains

In order to establish that 3B3 could bind selectively to endogenous AβOs, we characterized brain tissue sections from APP/PS1 Tg mice using array tomography, a technique that was designed for high-resolution histological characterization of synapses in brain samples^26,27,37^. Ribbons of 70 nm cortical serial sections were cut and bonded to glass slides, and immunostained for synaptic proteins and AβOs. The arrays were then fluorescently imaged to generate image stacks and reconstructed into 3D volumetric datasets for quantification.

**Figure 3:**
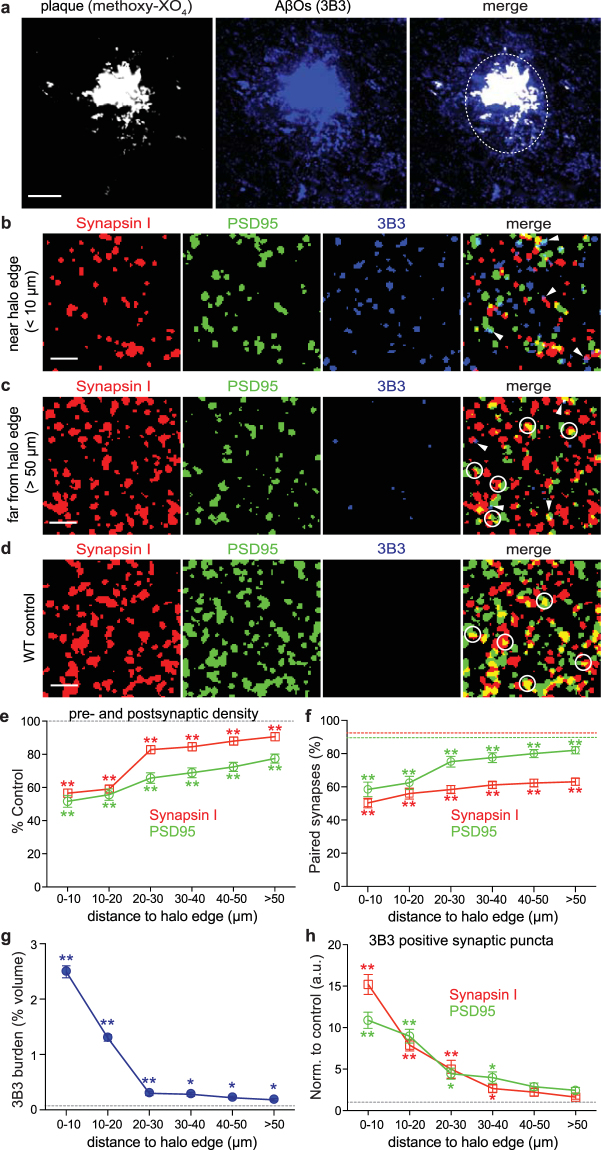
3B3 targets AβOs at synapses in APP/PS1 Tg mouse brains. (**a**) Projection of 17 array tomography sections shows that the dense core of the senile plaque (labeled with methoxy-XO_4_; white) is surrounded by an extended AβO halo (3B3 staining; blue). Dotted circle in merged panel indicates approximate edge of the AβO halo. (**b**–**d**) Presynaptic boutons (synapsin I; red), postsynaptic densities (PSD95; green) and AβOs (3B3; blue) were stained on ribbons of 70-nm sections from APP/PS1 Tg (**b** and **c**) and WT control tissue (**d**), and individual channels were merged to reveal co-localization between puncta. All images are maximum intensity z-projections of serial sections from array ribbons (12 sections projected in **b**, 14 sections projected in **c**, 20 sections projected in **d**). (**e**) Synaptic densities were quantified and normalized to age-matched WT control levels (grey dotted line) at 10 µm increments from the AβO halo edge (phantom plaques in controls were chosen randomly) to distances >50 µm from the nearest plaque (two-tailed Mann-Whitney *U* test, **p < 0.01). (**f**) Pairing analysis was performed to determine if synaptic puncta had a potential partner within 0.5 µm (examples circled in merged panels in **c** and **d**). More than 90% of pre- and postsynaptic puncta were paired in WT control brains (red and green dotted lines), whereas synaptic pairing was significantly impaired in APP/PS1 mouse brains (two-tailed Mann-Whitney *U* test, **p < 0.01). (**g**) The AβO burden (i.e. percentage of neuropil volume occupied by 3B3 staining) significantly increased as a function of distance to AβO halo edge (dotted line indicates averaged control level; two-tailed Mann-Whitney *U* test, *p < 0.05, **p < 0.01). (**h**) Percentage of synaptic puncta positive for AβO staining in APP/PS1 cortex (examples arrowhead-pointed in merged panels in **b** and **c**) remarkably increased by ~15 folds for synapsin I and ~11 folds for PSD95 near the halo edge compared with controls (dotted line), and then decreased monotonically in further distances (two-tailed Mann-Whitney *U* test, *p < 0.05, **p < 0.01). Data are shown as mean ± s.e.m. Scale bars: **a** = 10 µm; **b**, **c**, **d** = 2 µm.

To evaluate the distribution of AβOs in relation to senile plaques, the arrays were immunolabeled with 3B3 along with a fluorescent secondary antibody and plaques were stained with methoxy-XO_4_, a fluorescent marker that specifically stains plaques, tangles, and cerebrovascular amyloid^27,38^. We found that the dense cores of amyloid plaques stained positively along with an extended AβO halo on the surface of amyloid plaques, with substantial labeling well beyond the plaque dense core (Fig. 3a), similar to findings of Koffie *et al*.^27^ and Gaspar *et al*.^39^.

To understand the impact of AβOs on synapses, we assessed 15 plaques and over 15,000 synapses in APP/PS1 Tg mouse brains and over 25,000 synapses in age-matched WT control samples (for detailed case information and numbers, refer to Table 1). APP/PS1 Tg mice exhibited substantial reductions in both synapsin I and PSD95 immunostaining near the halo of oligomeric Aβ (Fig. 3b), compared to regions far from the plaques (>50 µm; Fig. 3c) and WT controls (Fig. 3d). The density of pre- and postsynaptic elements decreased progressively close to plaques in APP/PS1 Tg mice (Fig. 3e, red and green lines), whereas synaptic density did not vary at distances from a randomly placed phantom in WT cortex (Fig. 3e, dotted line). In the immediate vicinity of the AβO halo, significant reductions (44% for synapsin I and 47% for PSD95) in synaptic density were prominent compared with controls (Mann-Whitney *U* test, **p < 0.0001). The density of presynaptic boutons approached control levels (~83%) at distances of 20 to 30 µm from the halo edge, and continued to increase in volumes further than 50 µm away from the nearest plaque (Fig. 3e, red line). The density of PSD95 increased in an approximately linear fashion radiating out from halo edge and exhibited more prominent loss (compared with synapsin I), even in volumes far away from the plaque (Fig. 3e, green line), suggesting that the postsynaptic compartments, in contrast to their presynaptic partners, may be compromised to a greater extent throughout disease progression.

**Table 1:** Characteristics of mouse samples used in array tomography study.

*Diagnosis/Genotype*	Case#	Age (month)	Gender	Case Description	Plaques	Presynaptic puncta	Postsynaptic puncta	AβO puncta
APP/PS1	6058-bcx1	8.13	Female	Barrel cortex	5	7,599	6,724	326
APP/PS1	6059-bcx1	8.13	Female	Barrel cortex	5	6,792	6,165	293
APP/PS1	9289-bcx1	15.67	Female	Barrel cortex	5	4,766	4,103	372
C57 WT	4574	8.03	male	Barrel cortex	0	11,428	10,293	89
C57 WT	4575	8.03	male	Barrel cortex	0	9,857	9,053	63
C57 WT	C57-22	14.87	Female	Barrel cortex	0	8,238	8,364	75

WT – Wild-type.

A pairing analysis described previously^37^ was performed to determine whether any of the pre- or postsynaptic elements were left orphaned, i.e., whether each individual puncta had become spatially isolated from a potential connecting synaptic partner within 0.5 µm. In WT cortex, ~92% of synapsin I puncta and ~90% of PSD95 puncta were paired (Fig. 3f, red and green dotted lines). Conversely, synaptic pairing was remarkably impaired in APP/PS1 cortex, particularly at the AβO halo edge, where only 50% of synapsin I puncta and 58% of PSD95 puncta were paired (Fig. 3f, red and green lines). At distances far from the plaque (>50 µm), only ~63% of synapsin I puncta retained a synaptic connection, whereas 81% of PSD95 puncta were paired, although both were statistically reduced in comparison to WT controls (Mann-Whitney *U* test, **p < 0.001). This suggests that postsynaptic sites may degenerate earlier or faster than presynaptic boutons in the presence of AβOs in APP/PS1 Tg mice. Additionally, the absence of pairing reflects a reduced number of functional synapses, which likely alters neural network properties.

To test whether 3B3-positive oligomers might affect synapse loss, we assessed AβO burden in relation to the distance from the halo edge. Concentrations of AβOs, as determined by 3B3 labeling in Tg cortex were markedly higher near the halo edge compared with WT control levels (Fig. 3g). Moreover, the percentage of pre- and postsynaptic puncta that stained positive for AβOs increased drastically as a function of proximity to halo edge in APP/PS1 Tg brains (Fig. 3h).

We performed *post hoc* statistical correlations to examine the relationship between 3B3 burden and either synaptic densities, synaptic pairing, or the proportion of synapses positive for AβO staining, respectively. Both the density and pairing of pre- and postsynaptic elements revealed strong negative correlation with 3B3 burden (Spearman’s correlation coefficients, −0.926 for synapsin density, −0.900 for PSD density, −0.957 for synapsin pairing, −0.942 for PSD pairing; *p* < 0.01). Co-localization of synaptic puncta with AβOs was positively associated (Spearman’s correlation coefficients, 0.981 for 3B3-positive synapsin puncta, 0.959 for 3B3-positive PSD puncta; *p* < 0.01). These results provide additional strong support for the synaptotoxic role of AβOs in disease progression.

### 3B3 targets AβOs at synapses in human AD brains

**Figure 4:**
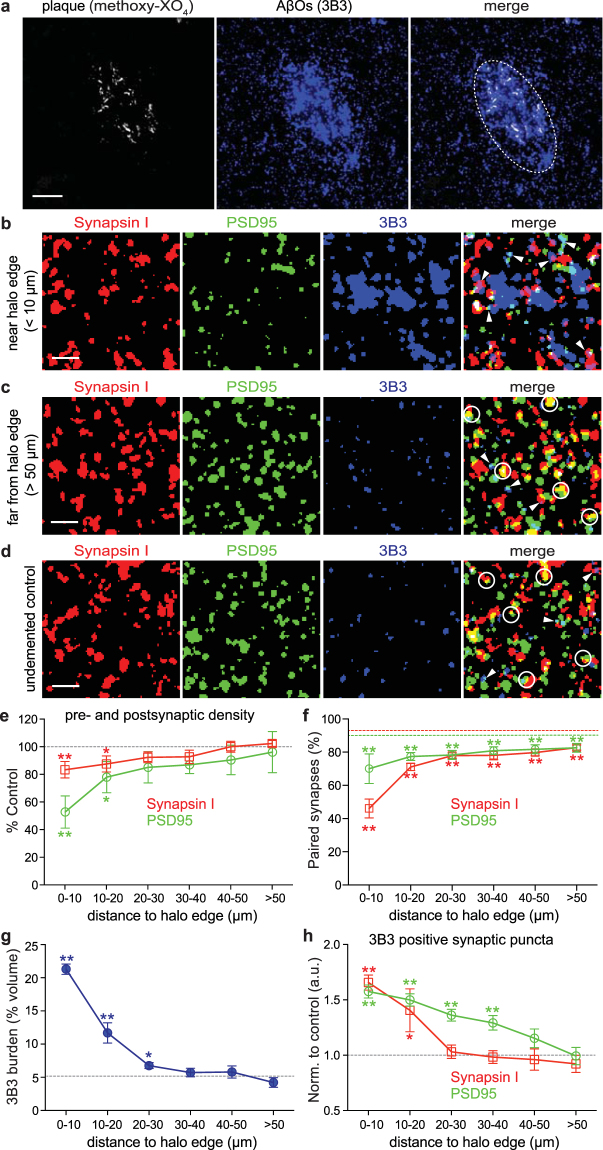
3B3 targets natural AβOs at synapses in human AD brains. (**a**) Projection of 15 array tomography sections shows that a diffuse-like plaque (labeled with methoxy-XO_4_; white) in human AD tissue is surrounded by a concentrated area of AβOs (3B3 staining; blue). Dotted circle in merged panel indicates approximate edge of the AβO halo determined by visual inspection. (**b**–**d**) Presynaptic boutons (synapsin I; red), postsynaptic densities (PSD95; green) and AβOs (3B3; blue) were stained on ribbons of 70-nm sections of human AD (**b** and **c**) and non-demented control cortices (**d**). Channels were merged to reveal co-localization between puncta. All images are maximum intensity z-projections of serial sections from array ribbons (16 sections projected in **b**, 18 sections projected in **c**, 25 sections projected in **d**). (**e**) Synaptic densities were quantified and normalized to control levels (grey dotted line) at 10 µm increments from the AβO halo edge (phantom plaques in controls were chosen randomly) spreading out to >50 µm from the nearest plaque (two-tailed Mann-Whitney *U* test, *p < 0.05, **p < 0.01). (**f**) More than 90% of pre- and postsynaptic puncta had a potential partner in non-demented control brains (red and green dotted lines indicate control averages), whereas synaptic pairing was substantially reduced in AD brains (two-tailed Mann-Whitney *U* test, **p < 0.01). (**g)** AβO burden represented by 3B3 staining shows a significant rise in the vicinity of the halo edge with a trend towards an increase between 0–30 µm approaching the AβO halo edge (dotted line shows averaged control level; two-tailed unpaired *t*-test, *p < 0.05, **p < 0.01). (**h**) Percentage of pre- and postsynaptic puncta positive for 3B3 burden (examples arrowhead-pointed in merged panels in **b**, **c** and **d**) significantly increased by ~60% for both synapsin I and PSD95 markers close to the AβO halo edge in AD brains compared with non-demented controls (dotted line indicates normalized control level; two-tailed Mann-Whitney *U* test, *p < 0.05, **p < 0.01). Data are shown as mean ± s.e.m. Scale bars: **a** = 10 µm; **b**, **c**, **d** = 2 µm.

To study AβO-targeting of synapses in human brain, we used tissue samples from three neuropathologically confirmed AD brains and three non-demented control brains, evaluating a total of 15 plaques and >10,000 synapses in AD samples and >20,000 synapses in control samples (for detailed case information and numbers, refer to Table 2). In contrast to dense-core plaques surrounded by an extended AβO halo in APP/PS1 cortex, human AD tissue exhibited non-cored diffuse-like plaques surrounded by a concentrated pool of 3B3 staining (Fig. 4a). Thus, the ‘halo’ edge in these AD tissue images was determined by visual inspection.

**Table 2:** Characteristics of human subjects used in array tomography study.

*Diagnosis/Genotype*	Case#	Age (year-old)	Gender	Case Description	Plaques	Presynaptic puncta	Postsynaptic puncta	AβO puncta
AD Human	1446	84	Male	B&B V/VI; probable by CERAD; ApoE 3/4	5	3,872	3,464	737
AD Human	1442	80	Female	B&B VI/VI; probable by CERAD; ApoE 4/4	5	3,615	3,073	694
AD Human	1418	84	Female	B&B VI/VI; definite by CERAD; ApoE 4/4	5	3,709	3,261	709
Control Human	1090	94	Male	Infarcts	0	7,942	7,729	417
Control Human	1190	88	Female	Strokes	0	8,374	8,023	482
Control Human	1619	82	Male	Diffuse hypoxic/ischemic injury	0	6,295	5,937	368

AD – Alzheimer’s disease; B&B – Braak and Braak staging system; CERAD – The Consortium to Establish a Registry for Alzheimer’s Disease; ApoE – Apolipoprotein E.

Human AD brains exhibited substantially diminished synapsin I and PSD95 staining, but prominently elevated 3B3 labeling immediately beyond the halo edge (Fig. 4b), compared with brain regions either far from the plaque cores (>50 µm; Fig. 4c) or in the non-demented controls (Fig. 4d). PSD95 density was reduced ~48% near the AβO halo, whereas reduction of synapsin I density in the same volumes was considerably lower at ~17%. Both measurements were statistically significant (Fig. 4e; Mann-Whitney *U* test, **p < 0.001). In general, synaptic density increased progressively from the edge of the AβO halo, reaching close to control levels in volumes more than 50 µm away from the halo edge. Synaptic pairing was also compromised, with the greatest pairing deficiencies occurring at the AβO halo edge for both pre- and postsynaptic elements (Fig. 4f).

AβO burden in non-demented controls was determined to be 5.3 ± 1.32% (mean ± s.d.; Fig. 4g; dotted line shows averaged burden level) volume occupied by 3B3 staining in tested cortical regions, lending support to the idea that AβOs are present in brain tissue from elderly individuals who do not yet exhibit measurable cognitive deficits^9,40–42^. AD brain tissue exhibited elevated 3B3 burden at distances of 0 to 30 µm away from the AβO halo, with the highest level (~21%) detected near the halo edge (Fig. 4g; blue line). Additionally, the percentage of 3B3-labeled synapses declined monotonically as a function of distance from the halo edge (Fig. 4h). Co-localization of synaptic puncta with AβO staining immediately outside of the halo edge was elevated significantly (by ~65% for synapsin I and ~60% for PSD95) compared with non-demented controls.

In human AD tissue, synaptic density and pairing were negatively correlated with 3B3 burden (Spearman’s correlation coefficients, −0.854 for synapsin density, −0.989 for PSD density, −0.959 for synapsin pairing, −0.975 for PSD pairing; *p* < 0.05), while co-localization of synaptic puncta with AβO was positively correlated with 3B3 burden (Spearman’s correlation coefficients, 0.824 for 3B3-positive synapsin puncta, 0.973 for 3B3-positive PSD puncta; *p* < 0.05). These results are consistent with our finding in APP/PS1 Tg brain tissue, providing a consistent AβO binding and selectivity profile for 3B3 in both Tg mouse and human AD brains.

## Discussion

Synapse loss is a fundamental characteristic of AD etiology that correlates strongly with cognitive decline^13,43–46^. Soluble Aβ oligomers are potent neurotoxins that have been implicated in memory loss, synaptic dysfunction and neurodegeneration, all of which increase progressively into late stages of AD^1,3,6,11,47,48^. Mounting evidence suggests that the selective neuronal vulnerability manifest in AD derives from specific AβO binding to high affinity receptors^3,6,49^ expressed by particular subtypes of neurons^1,50^, leading to disruption of synaptic signaling pathways^51–54^, blockade of long-term potentiation^1,55,56^, and alterations in spine morphology and density^27,37,47,48,51^. Thus, specific targeting of AβOs to prevent synaptic binding and consequent aberrant signaling should mediate highly effective AD treatment and prevention of cognitive decline.

We sought to develop a rapid, sensitive method for screening, identification and characterization of potential AβO-blocking antibody candidates, based on real-time calcium imaging. Calcium homeostasis is tightly maintained for proper neuronal functions, however, highly potent AβOs are known to elevate intracellular calcium specifically in cultured neurons^36,57,58^ and *in vivo*^35,36^. Because ratiometric calcium indicators allow simultaneous monitoring of [Ca^2+^]_i_ in a large population of cellular compartments in real time, changes in measured [Ca^2+^]_i_ reflect the extent of aberrant AβO neuronal signaling and/or protection by AβO-targeting antibodies. Recently we described a study of Tg2576 mice treated for 6 months with the anti-Aβ antibody aducanumab, which binds fibrillar Aβ but not monomer. The elevated calcium levels exhibited by Tg2576 mice were normalized by aducanumab to levels observed in WT mice, suggesting that aducanumab blockage of AβO activity may underlie the promising early AD clinical trial results^59^. Thus, the sensitive, real-time functional calcium assay described here appears useful for initial-stage anti-AβO drug screening and for more extensive *in vivo* efficacy characterization of potential AβO-directed therapeutics in animal models.

An important, essential feature of all experiments in this study was our deployment of AβO concentrations in the low nanomolar to picomolar range. These concentrations are comparable to AβO levels that likely exist in AD brain, and orders of magnitude lower than concentrations deployed in the vast majority of AβO-related studies^30,31^. Yet, these low levels of AβOs were capable of triggering substantial increases in intracellular calcium. Importantly, sub-nanomolar AβO concentrations induced [Ca^2+^]_i_ increases in a fraction of cultured neurons, likely the same sub-population capable of binding AβOs^6,34^. Immunodepletion of AβOs with the AβO-selective antibody 3B3 generated preparations that did not elevate neuronal [Ca^2+^]_i_, but attempted immunodepletion with heat-inactivated 3B3 did not abrogate AβO-triggered [Ca^2+^]_i_ increases. We confirmed these AβO-mediated effects on neuronal calcium *in vivo* with multiphoton microscopy imaging of layer 5 neurons specifically expressing the viral vector-encoded calcium imaging probe YC3.6. Topical application of AβOs to exposed cortical surfaces resulted in calcium overload in a fraction of neurons in WT mice, with imaging profiles very similar to profiles observed in Tg APP mice. Pretreatment of cortical surfaces with 3B3 prior to AβO application completely prevented calcium elevation in living animals, suggesting that immunotherapy with a human AβO-selective antibody such as ACU193^9,10^ could provide substantial therapeutic benefit by blocking AβOs and the synaptotoxic aberrant signaling they trigger.

The underlying mechanisms of AβO-induced neuronal calcium dysregulation remain to be elucidated. The data reported here support the idea that the sub-population of affected neurons express specific AβO receptors, most likely post-synaptic, that mediate downstream signaling. Many reports of putative AβO receptors have been published, but no consensus exists that any particular protein is the relevant AD-relevant AβO receptor^60^. Excessive calcium influx through activated *N*-Methyl-D-aspartate (NMDA) receptors has been implicated in AβO-dependent calcium elevation *in vivo*, and this deleterious alteration was effectively abolished by topical application of the NMDA receptor antagonist MK-801 to the brain surface of living animals. AβO-triggered decrease in dendritic spine density *in vivo* was also effectively blocked with MK-801 treatment^36^. Inhibition of P/Q- and N-type VGCCs prevented AβO-induced presynaptic calcium influx that specifically blocks brain-derived neurotrophic factor transport in axons^61^. In addition to promoting calcium entry from extracellular sources, AβOs also induced free calcium release from intracellular endoplasmic reticulum (ER) stores via ryanodine receptors (RyRs) and inositol 1,4,5-trisphosphate (IP_3_) receptors^62^. Dantrolene, an inhibitor of RyRs in particular, was shown to diminish Aβ load, normalize ER calcium signaling and ameliorate memory deficits in three different transgenic mouse models of AD^63–65^. Independent lines of evidence indicate that AβOs could potently elevate intracellular calcium levels by either stimulating production of IP_3_^66^ and/or recruiting mGluR5 at the synapse^67^. Determining which, if any of these pathways is linked to AD-relevant AβO activity will likely require identification of the *bona fide* high affinity, neuronal sub-type specific AβO receptor, and elucidation of its particular signaling pathways.

In addition to development of the combined *in vitro*/*in vivo* screening assay, we also deployed array tomography to detect AβO distribution and binding localization with specific synaptic elements at extremely high spatial resolution, using AβO-selective labeling with 3B3 in APP/PS1 Tg mouse and human AD brain tissue sections. We found that AβO were prominently detected within the plaques and formed a halo that surrounds the plaques. Immunolabeling of synapsin I and PSD95, pre- and postsynaptic proteins respectively, allowed us to observe that the density of synapsin I and PSD95 decreased progressively near the plaques, along with the percentage of paired synaptic partners. AβOs co-localized with both pre- and postsynaptic elements, and the co-localization was more likely to occur near plaques where AβO concentration was higher. Similar results were obtained for human AD tissue and for Tg mouse tissue, providing convincing evidence that specific AβO binding at synapses disrupts synaptic signaling and likely leads to synapse loss.

The most important implication of this study is that potent, AβO-selective immunotherapeutics such as ACU193, the human homolog of 3B3 studied here, should prove to be highly effective for treatment and prevention of AD. Although AβOs have been implicated as AD-relevant neurotoxins for nearly two decades, clinical trials over the past decade have tested only non-specific antibodies targeting non-toxic Aβ monomer and/or low toxicity Aβ fibrils. Some of these failed antibodies are also capable of binding AβOs, yet all of them failed because brain levels of monomer and fibril are substantially higher than levels of therapeutic antibody delivered to the brain at maximum dosing. In contrast, brain levels of AβOs are estimated to be 2,500-fold lower than Aβ monomer and >1 million-fold lower than plaque Aβ^9,68–74^ so that AβO blockade is likely to be more successful with brain antibody levels readily achieved with modest dosing. The attractive AβO-blocking profile of 3B3 described here presents a compelling rationale to focus future therapeutic development on compounds that directly block AβOs, their assembly, their specific receptors, or their deleterious downstream signaling.

## Methods

### Animals

The research protocols implemented in this study were approved by the Massachusetts General Hospital Institutional Animal Care and Use Committee. All experiments were performed according to institutional guidelines (Massachusetts General Hospital Animal Care and Use Committee) and in compliance with national guidelines (U.S. National Institutes of Health) for the use of experimental animals. Characteristics of APP/PS1 transgenic mice used for array tomography study are summarized in Table 1. WT C57BL/6 (Jackson Laboratory) males 3–4 months of age were used for *in vivo* experiments.

### Human subjects

Brains from human subjects with a neuropathological diagnosis of AD or non-demented age matched controls were obtained through the Massachusetts Alzheimer’s Disease Research Center (MADRC) that maintains a brain tissue bank at the Massachusetts General Hospital. Informed consent for brain autopsy was obtained from all subjects. The Massachusetts General Hospital Institutional Review Board approved the study protocol. All of the AD subjects fulfilled the National Institute on Aging-Reagan criteria for high likelihood of AD. All donor tissue was collected and stored by MADRC in accordance with local and National Institutional Review Board regulations. Characteristics of each subject are shown in Table 2.

### Array tomography

Array tomography was performed as previously described^26,75^. Cortical tissue samples (≈1 mm^3^) were dissected and fixed in 4% paraformaldehyde and 2.5% sucrose in 0.01 M PBS for 3 hours. After dehydration in ethanol, samples were infiltrated in LR White resin (Electron Microscopy Sciences) overnight at 4 °C before being polymerized at 53 °C. Embedded blocks were then cut into ribbons of sections (70 nm) on an Ultracut microtome (Leica) with a Jumbo Histo Diamond Knife (DiATOME).

After rehydration in 50 mM glycine in Tris-buffered saline (TBS) for 5 min, sections were blocked in 0.05% Tween and 0.1% bovine serum albumin in TBS for 5 min. Primary antibodies [rabbit anti-PSD95 (Cell Signaling Technology), goat anti-PSD95 (Abcam), rabbit anti-Synapsin I (Millipore), and mouse anti-Aβ oligomer (Acumen Pharmaceuticals Inc., 3B3)] were applied at 1:50 dilution in blocking buffer overnight. Secondary antibodies [donkey anti-rabbit Alexa Fluor 488 (Invitrogen), donkey anti-mouse Alexa Fluor 488 (Invitrogen), donkey anti-rabbit Cy5 (Jackson ImmunoResearch), donkey anti-mouse Cy3 (Jackson ImmunoResearch), and donkey anti-goat Cy3 (Jackson ImmunoResearch)] were diluted 1:50 in blocking buffer for 30 min. To stain plaques, 100 µM methoxy-XO_4_ was applied onto the ribbons for 10 min.

Images were obtained on 10 to 30 serial sections by using a Zeiss AxioImager Z2 fluorescence microscope (63×/1.2 Plan Apochromat oil objective) with automated imaging macros built in the AxioVision software. Several cortical sites were sampled per mouse/human subject.

Images were analyzed as previously described^75^ with ImageJ (NIH) and MATLAB (MathWorks) programs developed in house. Each set of images was converted to stacks, then aligned, cropped into volumes at selected distances from user defined detection borders with MultiStackReg and StackReg plug-ins [courtesy of B. Busse and Thevenaz *et al*.^76^]. An automated iterative threshold-based detection algorithm was applied to count labeled puncta appearing in more than one consecutive section from each channel and the output image stacks were further analyzed for co-localization and pairing between stained proteins with custom scripts written in MATLAB.

### Neuronal Culture

Primary neuronal cultures were prepared from cerebral cortices of embryonic day (E) 14–15 CD1 mouse embryos (Charles River Laboratories) as described elsewhere^77^ with modifications. Cortices were dissected out and mechanically dissociated using a Papain Dissociation System (Worthington Biochemical Corp.) according to the manufacturer’s protocol. The neurons were maintained in Neurobasal (Invitrogen) medium with 2% B27 supplement (Invitrogen), 2 mM Glutamax (Gibco), 100 U/mL penicillin (Gibco), and 100 g/mL streptomycin (Gibco) at 37 °C with 5% CO_2_ for 12–14 days or 21 days prior to experiment.

### Preparation of Synthetic AβOs

Synthetic AβOs were prepared from human Aβ_1–42_ (AnaSpec Inc.; Catalog # AS-20276) and control preparations were prepared from scrambled human Aβ_1–42_ (AnaSpec Inc.; Catalog # AS-25383) as described previously^29^ with modifications. Briefly, 1 mg synthetic Aβ_1–42_ was dissolved in 222 mL cold hexafluoro-2-propanol (Sigma-Aldrich) and the mixture was vortexed and incubated in a 37 °C water bath for 1 h. The solvent was then evaporated completely using a Speedvac for 20 min. The resulting peptide (appearing as a clear thin film) was stored at −20 °C overnight before being dissolved in fresh anhydrous dimethyl sulfoxide (DMSO; Life Technologies), providing a stock concentration of ~100 µM. The actual concentration of the stock Aβ solution was determined spectrophotometrically by measuring absorbance at 280 nm and computing according to the Beer-Lambert law. The stock was then diluted in 50 mL Ringer’s Solution (B. Broun Medical Inc.) at 37 °C to generate working solutions in the picomolar to nanomolar concentration range. Vehicle-only solutions for *in vitro* and *in vivo* imaging experiments were prepared using DMSO lacking any added peptide. The final AβO-containing solution was incubated for 15 min at 37 °C before use. The final concentration of DMSO in the Ringer’s solution was in the range of 0.0015–0.002% for cell culture experiments and 0.005–0.01% for the *in vivo* assays.

### Size exclusion chromatography of AβOs

AβOs were prepared as described above and incubated for 1 h at 37 °C. 1 mL of AβO solution was separated by SEC on single Superdex 75 columns (GE Healthcare) in 50 mM ammonium acetate (pH 8.5) at a flow rate of 0.5 mL/min, with an AKTA purifier 10 (GE Healthcare) and dialyzed against PBS. Aβ_42_ content was quantified in individual SEC fractions after dilution using the BAN50-BC05 two-site ELISA (Wako Chemicals) as suggested by the manufacturer’s instructions.

### Immunodepletion of AβOs

Recombinant Protein G beads (Dynabeads, Life Technologies) were pre-washed in Neurobasal medium and then added into 1 mL of 3 nM synthetic AβOs with 9 µg of testing antibody. The mixture was placed into Eppendorf tubes and rotated overnight at 4 °C. A magnet was then used to separate the beads from the media. The supernatant of immunodepleted AβOs was transferred into a clean tube after separation and stored at −80 °C prior to use.

### Aβ quantification

Human Aβ_42_ concentrations in the synthetic AβO samples were determined using BNT77-BC05 sandwich ELISA kit (Wako Pure Chemicals Industries, Japan), according to the manufacturer’s instructions.

### Live cell calcium imaging

At 12–14 days *in vitro* (DIV) or 21 DIV, neuronal cultures were incubated with 6 µM indo-1/AM dye (Invitrogen) and 2 µM sulforhodamine 101 (SR101; Life Technologies) for 45 min at 37 °C. Images were acquired using a 25× water immersion objective on an inverted Zeiss LSM 510 multiphoton microscope with a humidified environmental chamber that maintained temperature at 37 °C. Two-photon excitation was performed at 750 nm using a mode-locked Chameleon Ti:sapphire laser (Coherent, Inc) as the source. Ratiometric images were generated with the simultaneous acquisition using non-descanned detectors of two spectral channels: 390–465 nm and 480–522 nm. Multiple fields were acquired that included 300–400 cells per dish. After baseline calcium was obtained, the cultures were treated with antibody-immunodepleted AβOs or AβOs-alone by replacing half of the total volume of the media in the dish with oligomer-containing media for 45 min. The cultures were then re-imaged in the same areas in the dish.

### Stereotaxic intracortical virus injection

Intracortical injections of AAV2-CBA-YC3.6 virus were performed as described previously^78,79^. Mice were anesthetized with isoflurane (1–1.5%) and placed on a heated stereotaxic apparatus (Kopf Instruments). The surgical site was sterilized and a 2–3 mm incision was made in the scalp along the midline between the ears. Two burr holes were drilled in the skull with coordinates calculated from bregma (anteroposterior −1 mm and mediolateral ± 1 mm). Using a 10 µL syringe with 33-gauge sharp needle (Hamilton Medical), 3 µL of virus was injected at a depth of 1.2 mm in somatosensory cortex at a rate of 0.12 mL/min. One injection was carried out in each burr hole, the scalp was sutured after viral injection, and the animal was returned to the cage for recovery from anesthesia on a heating pad.

### Cranial window implantation and multiphoton imaging

Three to four weeks post viral injection, a cranial window was implanted by replacing a piece of skull above the somatosensory cortex with a 6 mm diameter cover glass as described previously^80,81^. The dura mater was gently removed prior to window installation for all the experiments. Texas Red dextran (12.5 mg/mL in 0.1 mL PBS; Molecular Probes) was injected intravenously to provide a fluorescent angiogram.

An Olympus BX61WI upright microscope (25×/1.05 XLPlan N water immersion objective) was used for *in vivo* imaging. A mode-locked DeepSee Ti:sapphire laser (Spectra Physics) generated two-photon excitation at 860 nm, and detectors containing three photomultiplier tubes (PMTs; Hamamatsu, Japan) collected emitted light in the range of 460–500, 520–560, and 575–630 nm^36^. Mice were placed on a heated microscope stage to maintain body temperature throughout the course of imaging. At least six cortical volumes (z-stacks with step interval of 2 µm, 212 × 212 µm^2^, depth of 200–300 µm) were imaged per animal at 3× magnification with a resolution of 512 × 512 pixels. The same cortical regions were always identified and reimaged before and after topical applications of AβOs to allow comparison of resting calcium within the same neuronal compartments. Cyan fluorescent protein (CFP) and yellow fluorescent protein (YFP) PMTs settings remained unchanged for calibration experiments and all imaging sessions. Laser intensity was adjusted as needed.

### Image processing and analysis

For indo-1 experiments in cell culture, single plane images of channels corresponding to calcium bound and unbound dye were obtained. For each channel, the background, corresponding to the mode of the image, was subtracted and a median filter was applied before dividing the emitted fluorescence intensity of the calcium bound channel by the unbound dye channel, thus creating a ratio-image. For YC3.6 *in vivo* experiments, single channels corresponding to CFP and YFP fluorescence were processed in the same way described for indo-1 to create a YFP/CFP ratio image. The same identified cell bodies and neurites were manually selected using the free hand tool in ImageJ, before and after a treatment. The selected regions of interest were then placed on the ratio image and measured.

To convert the measured ratios, either for indo-1 or YC3.6, into calcium concentrations, calibration experiments were performed in advance using Calcium Calibration Buffer Kits (Life Technologies). This allowed determination of the dynamic range (R_min_ and R_max_) for each indicator (indo-1: R_min_ = 0.661, R_max_ = 2.968; YC3.6: R_min_ = 1.19, R_max_ = 2.5). Ratios were then converted to actual calcium concentrations using values of R_min_, R_max_ and the dissociation constant (K_D_) of indo-1 (107.9 nM) or YC3.6 (278 nM) with standard ratiometric equations^82,83^.

Pseudocolored images were created in MATLAB based on the ratios of each indicator, and a calibrated color lookup table that ultimately converted the map to HSV colorspace. We used the ratio values to supply the hue and saturation (color) and the reference image to supply the value (intensity).

Comparison of before and after treatment was made for each imaging session. Data were presented as the fraction of cells/neurites with calcium overload. Because not all cells/neurites respond to the treatment, the fraction of cells/neurites with calcium overload may be considered a more sensitive way of evaluating an effect. Calcium overload was determined based on using a histogram approach and defining a threshold of two standard deviation (s.d.) above the mean in untreated/pre-imaging conditions^35^.

### Statistical analysis

Statistical analyses were performed in Prism (GraphPad), Excel (Microsoft) and custom programs written in MATLAB. Normality of data was assessed by using the Shapiro-Wilk test and D’Agostino & Pearson omnibus K2 test. Normally distributed datasets were analyzed by using two-tailed unpaired *t*-test. When normality could not be assumed or sample sizes were small, Mann-Whitney *U* test (for comparison of two groups), Wilcoxon signed-rank test (for comparison of matched-pairs) and Kruskal-Wallis test (for comparison of >2 groups) were applied. Spearman’s rho correlation was used to determine correlations. Calculated comparisons were at confidence interval 95%, i.e. *p* < 0.05 was considered significant. Samples were blinded for each analysis.
